# Arrest of Root Carious Lesions via Sodium Fluoride, Chlorhexidine and Silver Diamine Fluoride In Vitro

**DOI:** 10.3390/ma11010009

**Published:** 2017-12-22

**Authors:** Gerd Göstemeyer, Felix Schulze, Sebastian Paris, Falk Schwendicke

**Affiliations:** Department of Operative and Preventive Dentistry, Charité—Universitätsmedizin Berlin, Corporate Member of Freie Universität Berlin, Humboldt-Universität zu Berlin, and Berlin Institute of Health, Aßmannshauser Str. 4-6, 14197 Berlin, Germany; felix.schulze@charite.de (F.S.); sebastian.paris@charite.de (S.P.); falk.schwendicke@charite.de (F.S.)

**Keywords:** root caries, caries arrest, silver diamine fluoride, chlorhexidine, sodium fluoride

## Abstract

Objective: To compare the root carious lesion arrest of chlorhexidine (CHX) and silver diamine fluoride (SDF) varnishes and/or sodium fluoride rinses (NaF) in vitro. Background: Effective and easily applicable interventions for treating root carious lesions are needed, as these lesions are highly prevalent amongst elderly individuals. Methods: In 100 bovine dentin samples, artificial root carious lesions were induced using acetic acid and a continuous-culture *Lactobacillus rhamnosus* biofilm model. One quarter of each induced lesion was excavated and baseline dentinal bacterial counts assessed as Colony-Forming-Units (CFU) per mg. Samples were allocated to one of four treatments (*n* = 25/group): (1) untreated control; (2) 38% SDF or (3) 35% CHX varnish, each applied once, plus 500 ppm daily NaF rinse in the subsequent lesion progression phase; and (4) daily NaF rinses only. Samples were re-transferred to the biofilm model and submitted to a cariogenic challenge. After six days, another quarter of each lesion was used to assess bacterial counts and the remaining sample was used to assess integrated mineral loss (ΔZ) using microradiography. Results: ΔZ did not differ significantly between control (median (25th/75th percentiles): 9082 (7859/9782) vol % × µm), NaF (6704 (4507/9574) and SDF 7206 (5389/8082)) (*p* < 0.05/Kruskal–Wallis test). CHX significantly reduced ΔZ (3385 (2447/4496)) compared with all other groups (*p* < 0.05). Bacterial numbers did not differ significantly between control (1451 (875/2644) CFU/µg) and NaF (750 (260/1401)) (*p* > 0.05). SDF reduced bacterial counts (360 (136/1166)) significantly compared with control (*p* < 0.05). CHX reduced bacterial counts (190 (73/517)) significantly compared with NaF and control (*p* < 0.05). Conclusion: CHX varnish plus regular NaF rinses arrested root carious lesions most successfully.

## 1. Introduction

In many countries, the prevalence of root caries lesions among the elderly is high [1]. Several factors contribute to this. First, gingiva recession leads to root surfaces being exposed [2,3], which are more prone to bacterial adhesion and demineralization than enamel surfaces [4]. Second, older populations show reduced salivary flow and oftentimes impaired oral hygiene capacity. Third, the demographic development—with a larger share of the population becoming older and retaining a growing number of teeth—entails more surfaces both on individual and on population level being at high risk for root caries [1,5]. In addition, and as a result of this demographic trend, the population of elderly within nursing care is growing, with many patients being unable to perform regular oral hygiene (via tooth brushing) on their own, while tooth brushing by nursing staff is oftentimes inadequate [6,7,8,9].

While this calls for effective and easy to apply preventive regimens, there is also great need for managing existing root caries lesions [5]. Such management cannot be restricted to restorative care, as placed restorations have limited longevity [10], but also elderly patients are only limitedly mobile, with even non-conventional restorations being difficult to provide. Consequently, there is the need for non-restorative management options, allowing control of root caries lesions also in non-dental settings.

Several such management options are currently available. Daily provision of sodium fluoride rinses (NaF) has been shown to both prevent and arrest root caries lesions by preventing demineralization and facilitating remineralization of lesions [11,12,13]. Antibacterial substances such as chlorhexidine (CHX) have been found efficacious as well, especially in the absence of regular oral hygiene via tooth brushing [12,14,15]. Silver diamine fluoride (SDF) combines remineralizing and antibacterial effects and has been shown to prevent lesion induction as well as arrest root caries lesions [12,16,17].

Most of these strategies for arresting root caries lesions have been compared with conventional oral hygiene via tooth brushing (which is not available in all settings and individual predispositions) or no further or placebo treatment, but not often against each other. The present study aimed to compare the root caries lesion arrest of CHX and SDF varnishes and/or regular NaF rinses in an artificial biofilm model. Our primary null-hypothesis was that there was no sufficient difference in biofilm-induced mineral loss between groups. Our secondary null-hypothesis was that none of the treatments exerted any antibacterial effects on root caries lesions.

## 2. Results

**Figure 1:**
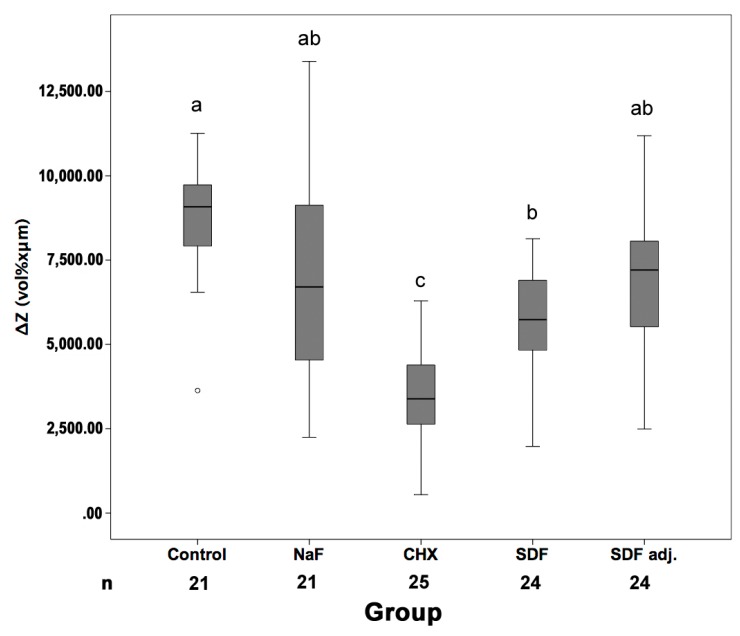
Mineral loss of root carious lesions in different groups. Samples were submitted to no treatment (Con), daily sodium fluoride (NaF) rinses only, chlorhexidine (CHX) or silver diamine fluoride varnishes plus daily NaF rinses. For SDF, artifact-adjusted (SDF_adj_) and unadjusted (SDF) values are reported. Line and box: Median and 25th/75th percentiles; whiskers: Range; circles: Outliers. Different superscript letters indicate statistically significant differences between groups (*p* < 0.05, Mann–Whitney/Bonferroni) (for example, NaF (superscript letter ab) is significantly different to CHX (subscript letter c) but not significantly different to the other groups (subscript letters ab, a or b)). n: Number of samples available for determining ΔZ.

From 100 samples, nine samples were lost during preparation for TMR (four in control group, four in NaF group, and one in SDF group) (Figure 1). ΔZ did not differ significantly between control, NaF and adjusted SDF (*p* > 0.05/Kruskal–Wallis). When not adjusting SDF values for possible artifacts, SDF showed significantly reduced mineral loss compared with control, but not NaF. CHX significantly reduced ΔZ compared with all other groups (*p* < 0.05).

Initial bacterial numbers within dentinal lesions did not differ significantly between groups (*p* = 0.674/Kruskal–Wallis). Bacterial numbers in lesions after conclusion of the experiment did not differ significantly between control and NaF (*p* > 0.05/Kruskal–Wallis, Table 1). SDF reduced bacterial counts compared with control (*p* < 0.05). CHX reduced bacterial counts compared with NaF and control (*p* < 0.05). The bacterial reduction did not differ between control, NaF and SDF, but was significantly larger in CHX than all other groups.

## 3. Discussion

Given the high prevalence of root carious lesions and the described demographic trend, there is great need for non-restorative approaches to arrest such lesions. While the accepted standard for such non-invasive management is regular plaque removal, this might not be effectively provided for all patients in all situations. Regular application of fluoride rinses alone or in combination with the application of a therapeutic varnish might be effective and require only limited efforts. We compared regimens of regular fluoride rinses with or without antibacterial and/or remineralizing varnishes for their effect on artificial bacterially-loaded root caries lesions. We found significant differences of integrated mineral loss between different groups, and thus reject our primary null-hypothesis. Moreover, we found CHX to have high antibacterial effects, thus also rejecting our secondary hypothesis.

Within the present investigation, we found daily application of NaF rinse alone to not significantly arrest caries lesions and prevent their further demineralization, which is in accordance with clinical findings [13] In contrast, NaF rinses were found to at least prevent dentin caries lesion induction in vitro [18,19]. However, the used caries models differed from that in our investigation, as lesions were created by pH cycling [18] or *Streptococcus mutans* biofilm [19]. Moreover, higher fluoride doses were used, while we used low concentrated fluoride rinses (as can be expected daily when using over-the-counter products), applied alone or in addition to SDF or CHX varnish. Application of higher fluoride concentrations might have had more pronounced arrest effects, given that root caries preventive effects of fluoride were found dose-dependent [18,19].

CHX varnish had the strongest effect on reduction of bacterial counts and prevention of further demineralization of the caries lesions. In contrast, another in vitro investigation did not find CHX to inhibit *L. rhamnosus* biofilm establishment and growth on root dentin [20]. However, within this study, CHX was applied as a rinse. It might be assumed that CHX, provided as a varnish as it was done in our study, exerted different effects: On the one hand, the pharmacokinetics differ, as effects of CHX will be present constantly given likely remnants within the dentin (in tubules or demineralized inter-tubular dentin). On the other hand, a CHX varnish could have created a diffusion barrier on top of the lesion, which could have blocked nutrition supply for dentinal bacteria. However, this sealant mechanism can be expected for SDF varnish as well, where only minimal antibacterial effects were found. Further studies are needed to elucidate the mechanism of the observed lesion arrest.

With regards to mineral loss, SDF did not arrest lesions compared with the untreated control group if the TMR analysis was adjusted for possible silver artifacts. When not performing any adjustments, a minimal beneficial effect was detected. Our findings are in contrast to that from other in vitro investigation where a caries arresting effect of SDF was detected [21,22]. However, the lesions in these investigations were induced by incubation either with *S. mutans* and *L. acidophilus* [21], or *S. mutans* or *A. naeslundii* [22]. Although several studies found SDF to have a strong antibacterial activity on cariogenic bacteria [21,22,23,24] the antibacterial activity on *L. rhamnosus* might be limited, leading to the limited arrest effects. Our findings with regards to CFU counts being only minimally affected by SDF supports this notion.

This study has several limitations. First, we measured the reduction of bacteria within dentinal lesions, but could not assess true lesion arrest as per microradiographically determined mineral loss difference. While, theoretically, we could have had tried and covered the initial lesion surface using nail varnish and then measure this baseline mineral loss after concluding the experiment, it was impossible to adhesively place any varnish on the relatively soft, bacterially infected dentin. As bacteria were to remain within this dentin, any fluid leakage would have allowed progression of this lesion, thereby distorting the measured mineral loss differences and lesion arrest. Future studies could aim and use other methods like transversal wavelength-independent microradiography [25] or µ-CT to longitudinally measure mineral loss. Second, varnish application was performed only once (as might be clinically desirable), and then rinsed off. That might underestimate the long-term removal of varnishes in situ by nutrition or mucosal contact. As a result, the varnish has likely remained not only within but also on top of the dentin, exerting some sealing-like effect, as discussed. This, however, might occur clinically in sheltered areas (i.e., those at risk), too, and could contribute to the effects of varnishes [26]. Third, our caries model was relatively aggressive and treated lesions highly infected; these lesions will not be found in all clinical situations. In general, the simulated oral conditions and the bacterial flora have limited external validity, as complex instead of single-species biofilms would be present, which are differently susceptible to antibacterial agents [27]. However, as these biofilms are variable between both individuals and settings [28], any model will have limited generalizability [29]. Clinically, lactobacilli are highly prevalent in root caries lesions, whereas other common cariogenic species such as *S. mutans* or *Actinomyces* play a minor role on root caries formation [28]. Given this strong association the use of *L. rhamnosus* within our study seems justified. Fourth, SDF treatment might distort microradiographic findings due to silver remnants artificially decreasing the measured mineral loss. We accounted for the resulting uncertainty appropriately, and found it to only limitedly impact on our findings. Fifth, we used bovine roots as substrate to simulate root dentin. As human and bovine dentin differ only limitedly with regards to their susceptibility for demineralization [30], this should not greatly impact on the transferability of our findings. However, it should be noted that the polished, flat surfaces used in our study deviate from the natural root surface anatomy, which is a necessary caveat to perform reliable microradiographic evaluation.

In conclusion, and within the limitations of the present study, a single application of a CHX varnish plus daily provision of a lowly concentrated NaF rinse was most successful in arresting simulated root caries lesion with regards to lesion demineralization and bacterial activity within the lesion. Clinical studies on root caries management should assess the effect of these agents in a real-life environment to gain evidence sufficient to give clinical recommendations.

## 4. Materials and Methods

### 4.1. Bacterial Culture

*Lactbacillus rhamnosus* GG (LGG, DSM 20021, DSZM, Braunschweig, Germany) was cultured for 48 h (37 °C) on de-Man-Rogosa-Sharpe agar (MRS, Difco, Franklin Lakes, NJ, USA). Cultures were inoculated into 100 mL MRS medium with 1% sucrose (MRS-S) and grown over night.

### 4.2. Specimen Preparation

**Figure 2:**
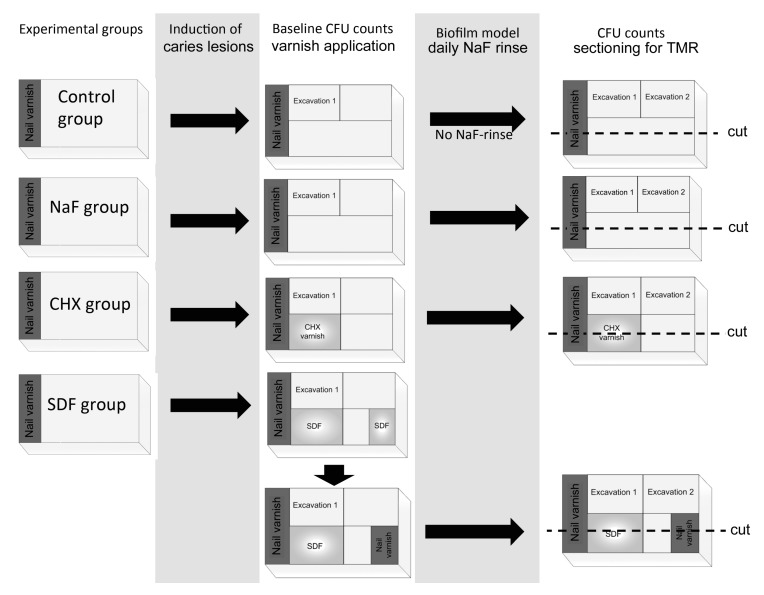
Experimental design for the different test groups. Caries lesions were induced by demineralization for two days and subsequently incubation with LGG. Baseline bacterial counts were obtained from excavated dentin and biofilms and varnishes were applied in the CHX and SDF groups. Cariogenic challenge was created within the biofilm model for six days with additional daily NaF-rinse application in all groups except the control group. CFU counts of carious dentin and demineralization in different areas of the samples was assessed by TMR.

Thirty bovine incisors of the second dentition where obtained from a local slaughterhouse (VION, Bad Bramstedt, Germany, vionfoodgroup.com). Extraction of the teeth was performed after slaughtering under supervision of the local veterinary. From the roots of the teeth, 100 dentin specimens (5 × 3 × 2 mm, Figure 2) were prepared (Band Saw Exakt 300 CL, Exakt Apparatebau, Norderstedt, Germany), ground flat (LaboPol 25, Struers, Ballerup, Denmark/Willich, Germany) and polished (abrasive paper SiC, P 1000–4000, Buehler, Düsseldorf, Germany). Twenty-five specimens per group were transferred into a silicone mold and embedded within acrylic resin (Technovit 4071, Heraeus Kulzer, Hanau, Germany). A 1 × 3 mm reference strip was covered with nail varnish (Long Lasting Nail Colour, Rival de Loop, Berlin, Germany), leaving an uncovered dentin surface of 4 × 3 mm. The resulting four carrier bars were sterilized (121 °C, 2.1 bar, 20 min, Tuttnauer 3870 ELV, Biomedis, Gießen, Germany).

### 4.3. Induction of Root Caries Lesions

First, root dentin samples were predemineralized in 1 L of a demineralizing solution (pH 5.0, 37 °C) containing 50 mM acetic acid, 3 mM CaCl_2_ × H_2_O, 3 mM KH_2_PO_2_ and 6 mM methyl-hydroxydiphosphonate for 2 days [31]. The pH of the solution was monitored daily (InLab micro, Mettler-Toledo, Giessen, Germany) and if necessary adjusted using HCl (Roth, Karlsruhe, Germany) or 10 M KOH. Samples were then incubated with overnight cultures of LGG in MRS-S for 24 h to induce bacterially contaminated lesions. Biofilms were removed from the surface areas of each specimen with a sterile scalpel. To allow enumeration of baseline bacterial load of induced lesions, dentin from one quarter of the lesion was now excavated using sterile rose-head burs and the net weight of excavated dentin assessed (Analytical Plus, Ohaus, Nänikon, Switzerland). Dentin was dissolved in 1 mL NaCl (0.9%), vortex mixed, and plated on MRS agar in various dilutions (10^2^–10^4^). Agar plates were cultured at 37 °C and 5% CO_2_ (CO_2_ Incubator, Heraeus Kulzer, Hanau, Germany). After 48 h, colony forming units per µg dentin (CFU/µg) were enumerated.

### 4.4. Treatments

Samples with induced lesions were submitted to one of four treatments (*n* = 25 per group): (1) No treatment (control); (2) 38% SDF (Riva Star, SDI, Baywater, Australia) or (3) 35% CHX (EC 40, Biodent, Nijmegen, The Netherlands) varnishes applied once, plus daily rinses of 500 ppm NaF rinses (Charité pharmacy, Berlin, Germany) during the subsequent second cariogenic biofilm challenge; or (4) only daily NaF rinses. Varnishes were applied with sterile application tips (roundtip applicator regular, Henry Schein, Melville, NY, USA) for 3 min. To simulate the removal of the varnishes from dentin surfaces by mastication or oral hygiene, varnishes were rinsed off with sterile aqua dest. at a temperature of 37 °C for 10 min.

### 4.5. Second Cariogenic Biofilm Challenge

The samples were transferred to a computer-controlled, multi-station continuous-culture biofilm model [32,33] with four different chambers (one carrier bar per chamber) at 100% humidity and 37 °C (Venticell 404 incubator, MMM Medcenter, Planegg, Germany). Nutrition, saliva, bacteria and NaF rinse were provided by peristaltic multi-channel pumps (MS/CA, ISMATEC, Wertheim, Germany).

To simulate a second cariogenic challenge, a coordinated sequence of nutritional medium and artificial saliva was provided. Each morning, carrier bars of all groups were inoculated with 5 mL cultures of LGG (approximately 7 × 10^6^ CFU/mL) for 15 min. Groups were then provided with pulses of MRS-S (1 mL/min for 15 min). Ten min after each sucrose pulse, modified defined mucin medium (DMM) [34] was provided at 1 mL/min for 15 min. This sequence was repeated at total of eight times daily. For all expect the control group, 8.5 mL 500 ppm NaF was provided immediately after the last daily MRS-S provision, followed by another 15 min DMM (1 mL/min) 10 min after the NaF pulse. An overnight resting period of 6 h was simulated. Overall, cultivation was performed for 6 days.

### 4.6. Bacterial Analysis and Microradiography

To determine the bacterial load after the second cariogenic challenge, the second third of the lesion was excavated as described and CFU/μg assessed. The remaining third of each specimen was cut perpendicularly to the surface into thin sections (Band Saw Exakt 300 cl) and ground to a thickness of 100 μm (Exakt Mikroschleifsystem 400 CS, Exakt Apparatebau). Before transferring into the X-ray source for transversal microradiography, specimens were imbibed in 99% ethylene glycol (Sigma Aldrich, Steinheim, Germany). Microradiographs were obtained by a nickel-filtered copper X-ray source (X-ray tube PW2213/20, Panalytical, Kassel, Germany, X-ray generator PW 1730/10, Philips, Eindhoven, The Netherlands) operating at 20 kV and 10 mA with an exposure of 10 s. Films (Fine 71337, Fujifilm, Tokyo, Japan) were developed according to manufacturer’s recommendation under standardized conditions. A digital-image-analyzing system (CFW 1312M, Scion, Frederick, MD, USA) interfaced with a universal microscope (CCD-video camera module XC 77 CE, Sony, Tokyo, Japan) and a personal computer (TMR for Windows, Version 2.0.27.2, Inspektor Research, Amsterdam, The Netherlands) was used to analyze lesions. During the preparation for TMR analysis, 9 of the 100 specimens were lost and could not be further analyzed.

As silver particles in SDF could affect micradiographically determined mineral loss, we adjusted ΔZ in the SDF group as follows: After the initial demineralization using acetic acid, one half of a 1 mm strip of each surface was covered with nail varnish. The other half of the strip was treated with SDF as described and also covered. When analyzing mineral loss after conclusion of the experiment, we evaluated the mineral loss difference in the induced initial lesions with and without SDF treatment. As a certain proportion of SDF particles might have been likely rinsed off in the experiment, but fully retained in the treated and nail-varnish covered reference area, the determined mineral loss difference was assumed to be the maximal artifact. Measured mineral loss values in the SDF were adjusted for this artifact, and adjusted and unadjusted values reported.

## 5. Statistical Analysis

Statistical analysis was performed with SPSS 22 (IBM, Armonk, NY, USA). Normal distribution was checked using the Shapiro–Wilk test. Differences in ΔZ and bacterial counts between different treatment groups were analyzed using two-sided Kruskal–Wallis and Mann–Whitney-U test, with Bonferroni correction for multiple testing and resulting alpha-inflation. The level of significance was set at *p* < 0.05.

## Figures and Tables

**Table 1:** Bacterial numbers in dentinal lesions (at baseline and after biofilm challenge) and bacterial reduction compared with baseline (before treatment).

Treatment	Bacterial Counts (CFU/μg)		Reduction
	(Baseline)	(After Biofilm-Challenge)	
Con	1721 (977/2671)	1451 (827/2645) ^a^	−17% (−57/120%) ^a^
NaF	1231 (458/5185)	750 (260/1401) ^ab^	−8% (−96/178%) ^a^
CHX	929 (417/3530)	190 (73/517) ^c^	−87% (−98/−68%) ^b^
SDF	1680 (625/4058)	360 (136/1166) ^bc^	−75% (−95/8%) ^a^

Samples were submitted to no treatment (Con), daily sodium fluoride (NaF) rinses only, chlorhexidine (CHX) or silver diamine fluoride (SDF) varnishes plus daily NaF rinses. Colony forming units per μg dentin (CFU/μg) and reductions (in percent compared with baseline) are reported as median (25th/75th percentiles). Different superscript letters indicate statistically significant differences between groups (*p* < 0.05, Mann–Whitney/Bonferroni) (for example, for reduction in CFU counts, CHX (subscript letter b) is significantly different than all other groups (subscript letter a)). *n* = 25/group.

## References

[1] Rodrigues J.A., Lussi A., Seemann R., Neuhaus K.W. (2011). Prevention of crown and root caries in adults. Periodontology 2000.

[2] Griffin S.O., Griffin P.M., Swann J.L., Zlobin N. (2004). Estimating rates of new root caries in older adults. J. Dent. Res..

[3] Bernabe E., Sheiham A. (2014). Age, period and cohort trends in caries of permanent teeth in four developed countries. Am. J. Public Health.

[4] Hoppenbrouwers P.M., Driessens F.C., Borggreven J.M. (1986). The vulnerability of unexposed human dental roots to demineralization. J. Dent. Res..

[5] Petersen P.E., Yamamoto T. (2005). Improving the oral health of older people: The approach of the WHO global oral health programme. Community Dent. Oral Eepidemiol..

[6] Peltola P., Vehkalahti M.M., Wuolijoki-Saaristo K. (2004). Oral health and treatment needs of the long-term hospitalised elderly. Gerodontology.

[7] Zenthofer A., Rammelsberg P., Cabrera T., Hassel A.J. (2014). Increasing dependency of older people in nursing homes is associated with need for dental treatments. Neuropsychiatr. Dis. Treat..

[8] Hiraishi N., Yiu C.K., King N.M., Tagami J., Tay F.R. (2010). Antimicrobial efficacy of 3.8% silver diamine fluoride and its effect on root dentin. J. Endod..

[9] Tan H.P., Lo E.C. (2014). Risk indicators for root caries in institutionalized elders. Community Dent. Oral Eepidemiol..

[10] Lo E.C.M., Luo Y., Tan H.P., Dyson J.E., Corbet E.F. (2006). Art and conventional root restorations in elders after 12 months. J. Dent. Res..

[11] Petersson L.G. (2013). The role of fluoride in the preventive management of dentin hypersensitivity and root caries. Clin. Oral Investig..

[12] Wierichs R.J., Meyer-Lueckel H. (2015). Systematic review on noninvasive treatment of root caries lesions. J. Dent. Res..

[13] Heijnsbroek M., Paraskevas S., Van der Weijden G.A. (2007). Fluoride interventions for root caries: A review. Oral Health Prev. Dent..

[14] Slot D.E., Vaandrager N.C., Van Loveren C., Van Palenstein Helderman W.H., Van der Weijden G.A. (2011). The effect of chlorhexidine varnish on root caries: A systematic review. Caries Res..

[15] Autio-Gold J. (2008). The role of chlorhexidine in caries prevention. Oper. Dent..

[16] Tan H.P., Lo E.C., Dyson J.E., Luo Y., Corbet E.F. (2010). A randomized trial on root caries prevention in elders. J. Dent. Res..

[17] Zhang W., McGrath C., Lo E.C., Li J.Y. (2013). Silver diamine fluoride and education to prevent and arrest root caries among community-dwelling elders. Caries Res..

[18] Garcia-Godoy F., Flaitz C., Hicks J. (2014). Role of fluoridated dentifrices in root caries formation in vitro. Am. J. Dent..

[19] Fernandez C.E., Tenuta L.M., Cury J.A. (2016). Validation of a cariogenic biofilm model to evaluate the effect of fluoride on enamel and root dentine demineralization. PLoS ONE.

[20] Zheng C.Y., Wang Z.H. (2011). Effects of chlorhexidine, listerine and fluoride listerine mouthrinses on four putative root-caries pathogens in the biofilm. Chin. J. Dent. Res..

[21] Mei M.L., Chu C.H., Low K.H., Che C.M., Lo E.C. (2013). Caries arresting effect of silver diamine fluoride on dentine carious lesion with *S. mutans* and *L. acidophilus* dual-species cariogenic biofilm. Med. Oral Patol. Oral Cir. Bucal.

[22] Chu C.H., Mei L., Seneviratne C.J., Lo E.C. (2012). Effects of silver diamine fluoride on dentine carious lesions induced by *Streptococcus mutans* and *Actinomyces naeslundii* biofilms. Int. J. Paediatr. Dent..

[23] Knight G.M., McIntyre J.M., Craig G.G., Mulyani, Zilm P.S., Gully N.J. (2009). Inability to form a biofilm of *Streptococcus mutans* on silver fluoride- and potassium iodide-treated demineralized dentin. Quintessence Int..

[24] Mei M.L., Li Q.L., Chu C.H., Lo E.C., Samaranayake L.P. (2013). Antibacterial effects of silver diamine fluoride on multi-species cariogenic biofilm on caries. Ann. Clin. Microbiol. Antimicrob..

[25] Thomas R.Z., Ruben J.L., de Vries J., ten Bosch J.J., Huysmans M.C.D.N.J.M. (2006). Transversal wavelength-independent microradiography, a method for monitoring caries lesions over time, validated with transversal microradiography. Caries Res..

[26] Al Dehailan L., Martinez-Mier E.A., Lippert F. (2016). The effect of fluoride varnishes on caries lesions: An in vitro investigation. Clin. Oral Investig..

[27] Mei M.L., Chu C.H., Lo E.C., Samaranayake L.P. (2013). Preventing root caries development under oral biofilm challenge in an artificial mouth. Med. Oral Patol. Oral Cir. Bucal.

[28] Preza D., Olsen I., Aas J.A., Willumsen T., Grinde B., Paster B.J. (2008). Bacterial profiles of root caries in elderly patients. J. Clin. Microbiol..

[29] Tang G., Yip H.K., Cutress T.W., Samaranayake L.P. (2003). Artificial mouth model systems and their contribution to caries research: A review. J. Dent..

[30] Lippert F., Churchley D., Lynch R.J. (2015). Effect of lesion baseline severity and mineral distribution on remineralization and progression of human and bovine dentin caries lesions. Caries Res..

[31] Buskes J.A., Christoffersen J., Arends J. (1985). Lesion formation and lesion remineralization in enamel under constant composition conditions. A new technique with applications. Caries Res..

[32] Sissons C.H., Cutress T.W., Hoffman M.P., Wakefield J.S. (1991). A multi-station dental plaque microcosm (artificial mouth) for the study of plaque growth, metabolism, pH, and mineralization. J. Dent. Res..

[33] Schwendicke F., Dorfer C., Kneist S., Meyer-Lueckel H., Paris S. (2014). Cariogenic effects of probiotic lactobacillus rhamnosus gg in a dental biofilm model. Caries Res..

[34] Wong L., Sissons C. (2001). A comparison of human dental plaque microcosm biofilms grown in an undefined medium and a chemically defined artificial saliva. Arch. Oral Biol..

